# Religiosity, spirituality in relation to disordered eating and body image concerns: A systematic review

**DOI:** 10.1186/s40337-015-0064-0

**Published:** 2015-08-15

**Authors:** Daniel Akrawi, Roger Bartrop, Ursula Potter, Stephen Touyz

**Affiliations:** School of Medicine, University of Western Sydney, Campbelltown, NSW Australia; Discipline of Psychiatry, Sydney Medical School- Northern, St Leonards, NSW Australia; Department of English, University of Sydney, Sydney, NSW Australia; Clinical Psychology Unit, School of Psychology, University of Sydney, Sydney, NSW Australia; Blacktown/Mt Druitt Clinical School, Blacktown Hospital, Sydney, NSW 2148 Australia

**Keywords:** Religiosity, Religion, Spirituality, Disordered Eating, Eating Disorder, Body Image

## Abstract

**Objective:**

This systematic review aims to critically examine the existing literature that has reported on the links between aspects of religiosity, spirituality and disordered eating, psychopathology and body image concerns.

**Method:**

A systematic search of online databases (PsycINFO, Medline, Embase and Web of Science) was conducted in December 2014. A search protocol was designed to identify relevant articles that quantitatively explored the relationship between various aspects of religiosity and/or spirituality and disordered eating, psychopathology and/or body image concerns in non-clinical samples of women and men.

**Results:**

Twenty-two studies were identified to have matched the inclusion criteria. Overall, the main findings to emerge were that strong and internalised religious beliefs coupled with having a secure and satisfying relationship with God were associated with lower levels of disordered eating, psychopathology and body image concern. Conversely, a superficial faith coupled with a doubtful and anxious relationship with God were associated with greater levels of disordered eating, psychopathology and body image concern.

**Discussion:**

While the studies reviewed have a number of evident limitations in design and methodology, there is sufficient evidence to make this avenue of enquiry worth pursuing. It is hoped that the direction provided by this review will lead to further investigation into the protective benefits of religiosity and spirituality in the development of a clinical eating disorder. Thus a stronger evidence base can then be utilised in developing community awareness and programs which reduce the risk.

## Review

With the rising prevalence of disordered eating [1, 2], and the detrimental health outcomes that often accompany it, research examining factors associated with disordered eating is now in need of urgent address. Despite the extensive literature describing the benefits of religiosity and spirituality in other domains of mental health, disordered eating has received less attention [3–8]. In fact, recent systematic reviews on religiosity, spirituality and mental health [9, 10] fail to take disordered eating into account.

A disordered eating continuum of severity exists, encompassing the absence of disordered eating behaviours and psychopathology, to moderate levels of disordered eating pathology, and eventually full-blown eating disorders [11–14]. Those who suffer from disordered eating pathology as well as those with body image concerns are at a greater risk of developing a clinical eating disorder [15–17]. Thus the benefits of religiosity and spirituality during non-clinical stages would offer valuable insight and be of greater use in the prevention of full-blown eating disorders.

The multidimensional nature of religiosity and spirituality inherently leads to a lack of consensus in defining these distinct yet overlapping concepts within literature. Consequently, most studies have aggregated religiosity and spirituality into one concept [6]. For the purpose of this systematic review, religiosity will be defined as a system of organised beliefs, practices, rituals and symbols designed to facilitate closeness to the transcendent, whereas spirituality will be defined as the personal quest for understanding answers to ultimate questions about life, meaning, and a relationship with the transcendent [18]. However these definitions should be taken with caution, as many constructs in the review will have overlapping features.

Religiosity and spirituality may have a positive, negative or non-significant influence on disordered eating, psychopathology and body image concerns. Religion often provides resources for coping with stressful events, as well as providing a sense of meaning and purpose through these times [10]. Religious and spiritual principles may also form a basis of self-worth which oppose societal standards of body image [19, 20]. In contrast, there are historical links between anorexia and asceticism, with self-starvation used as a means of achieving sanctity [21]. However, a fourth scenario may exist with different aspects of religiosity and spirituality being linked in differing ways to disordered eating, psychopathology and body image concerns. In addition to correlation analyses, it is hoped that information about moderation and mediation will offer greater insight into the pathways between risk factors and disordered eating, psychopathology and body image concerns.

 In a 2008 review of body image, disordered eating and religion, Boyatzis and Quinlan [22] found that most indices of religiosity related in healthy ways to women’s body image and eating behaviours, but concluded that methodological, design and sampling variables compromised the clinical value of the findings. One of the conclusions suggests that ‘scholarly attention must be paid to these dynamics between women’s religiosity and body image and eating behaviour’ [22] [p206]. The current systematic review has been undertaken in the spirit of these suggestions, with an aim of updating and expanding on their work. Their inclusion criteria consisted of published qualitative and quantitative studies exploring both clinical and non-clinical samples of women. The current review aims to utilise a more rigorous methodological system to search the literature to ensure a lower degree of selection bias. Therefore our systematic review will only include quantitative studies exploring non-clinical samples of women and/or men. Clinical samples will be excluded due to the variability emanating from their mixed participant characteristics. These include differing diagnoses, and the veracity thereof, as well as those currently in treatment as opposed to those not in treatment, and those closer to recovery in contrast to those in the acute stage. The complexities inherent in the multitude of variables may confound the outcome of this systematic review, and therefore to ensure homogeneity, only non-clinical samples will be chosen.

### Rationale for current review

This systematic review aims to critically examine the existing literature that has reported on the relationship between aspects of religiosity, spirituality and disordered eating, psychopathology and body image concerns. We aim to do this by updating and expanding on the review conducted by Boyatzis and Quinlan [22]. It is hoped that results emanating from this review will provide valuable directions for future clinical research.

## Method

### Search protocol

A search protocol was designed before the systematic literature search was commenced. The pre-determined inclusion criteria included published studies written in English which focused on non-clinical samples of women and/or men. The protocol required papers to quantitatively explore the relationship between various aspects of religiosity and/or spirituality and disordered eating, psychopathology and/or body image concerns.

Exclusion criteria for the articles comprised: (1) written in a language other than English; (2) books, dissertations, case studies, systematic reviews, conference abstracts, editorials or historical notes; (3) use of a clinical sample (4) evaluation of treatment/intervention/therapy; (5) body image concerns related to cancer or surgical outcome; (6) absence of data or statistical analysis; (7) absence of quantitative measures relating to aspects of religiosity or spirituality; (8) absence of quantitative measures of disordered eating, psychopathology or body image concerns (9) no exploration of the relationship between aspects of religiosity or spirituality and disordered eating, psychopathology or body image concerns.

### Search strategy

A systematic literature search of published studies was conducted in December 2014. The electronic databases of PsycINFO (1806-present), Medline (1946-present) and Embase (1974-present), accessed through Ovid and Web of Science accessed through Thomson Reuters were used. The databases were searched using different combinations of the following search terms in both subject headings and text words/titles: (Religion OR Religiousness OR Religiosity OR Spirituality OR Faith) AND (Eating Disorders OR Disordered Eating OR Anorexia Nervosa OR Bulimia Nervosa OR Binge Eating OR Eating disorders not otherwise specified OR Body Image). All subject headings were exploded in order to expand the search for possible studies.

### Selection of studies

**Fig. 1:**
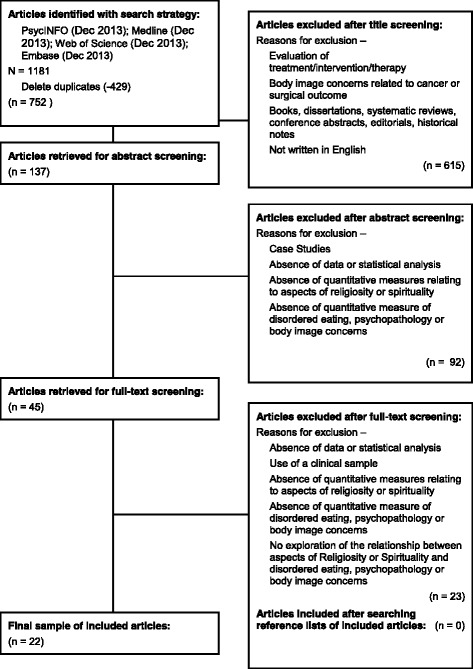
Flow diagram of the article exclusion process

1181 articles were retrieved from searching through the electronic databases. The flow diagram of the article exclusion process is presented in Fig. 1 (Appendix 1). After reviewing the results, it was found that 429 of the articles were duplicates, leaving a total of 752. Screening through the titles of the articles, 615 articles were excluded based on the exclusion criteria. Furthermore, another 92 articles were excluded after abstract screening. This left 45 articles for a full-text screen, following which 23 articles were excluded, culminating in the final 22 articles. At each step of the process, a second reviewer screened through the results to reduce selection bias. A manual search of the reference lists of the final 22 articles was also conducted, however no additional papers were found which matched the inclusion criteria.

### Quality assessment

The included studies were critically appraised using an amended version of the Quality Index (Appendix 2) [23]. The subscales of the index include reporting, external validity, internal validity and power. Specifically external validity explored the representativeness of the sample while internal validity examined the validity and reliability of the measures used, as well as adjustment for confounding. Overall, the amended quality index contained 14 items (refer to Appendix 1) which were scored 1 (Yes) or 0 (No/Unable to Determine). Two reviewers (DA & RB) undertook the critical appraisal initially independently. Results were reviewed and a common quality index score for each paper was decided upon when differences existed. Furthermore, each study was graded according to a hierarchy of evidence through a criteria used by Becker et al. (Appendix 3) [24]. Each article was given a grade ranging from I to IV based on the design and quality of the study.

### Data extraction and synthesis

Data was extracted by the first reviewer (DA) for each publication and placed into two summary tables. Table 1 (Appendix 4) includes variables such as study design and focus along with sample characteristics. Table 2 (Appendix 5) contains information about the measures used, results and quality assessment scores, and is divided into two sections. The first section lists articles that explore disordered eating and psychopathology, while the second section lists articles that explore body image concerns. The articles that explore all three concepts have their corresponding results displayed in separate sections. A second reviewer (RB) also checked the data entered into the tables to reduce error and bias.

**Table 1:** Summary of reviewed studies

Author (Year) (Country)	Study design	Sample size/ characteristic	Focus of study
Boisvert et al. (2013) (Canada) [38]	Cross-Sectional study	N = 591 women from Alberta, Canada. Mean age = 44.32. White (90.19 %), Asian (4.74 %), Hispanic (2.54 %), Aboriginal (2.54 %).	Investigated relationships between ethnicity, spirituality, religiosity, body shame, BMI, age and eating disorder symptomatology in women.
Boisvert et al. (2012) (Canada) [37]	Cross-Sectional study	N = 603 men in Alberta, Canada. Mean age = 42.33. White (86.0 %%), Asian (6.06 %%), Hispanic (2.5 %), Aboriginal (2.4 %).	Investigated ethnicity, spirituality, body shame, body mass index (BMI) and age as risk factors for eating disorder symptomatology in men.
Boyatzis et al. (2007) (USA) [25]	Random Assignment, pretest-posttest design	N = 125 women enrolled in a private university. Protestant (41 %), Catholic (34 %), Jewish (11 %), Agnostic or Atheist (13 %).	Tested whether college women's body image would improve after reading religious and spiritual affirmations about their bodies.
Boyatzis et al. (2006) (USA) [35]	Cross- Sectional study	N = 151 women enrolled in a private university and recent graduates. Protestant (40–60 %), Roman Catholic (30–40 %), Other (10–20 %).	Examined relations between women’s total Quest scores and body image and eating behaviour.
Buser (2013) (USA) [34]	Cross-Sectional study	N = 605 female undergraduate students from a secular university. Mean age = 22.8. Catholic (44.6 %), Protestant (15 %), Jewish (6.1 %), Buddhist (0.7 %), Hindu (0.8 %), Universalist (0.5 %), Mormon (0.2 %), Muslim (1 %), Atheist (2.8 %), Agnostic (4.3 %), Other (9.6 %), No religious affiliation (14 %).	Examined the influence of 5 types of spiritual coping on bulimic symptoms.
Feinson et al. (2012) (Israel) [41]	Cross-Sectional study	N = 790 women. Mean age = 44. Ultra-Orthodox (33 %), Orthodox (23 %), Traditional (21 %), Secular (23 %).	Explored religious observance and its presumed protective role for ultra-Orthodox women.
Forthun et al. (2003) (USA) [29]	Cross-Sectional study	N = 876 women from a large Southwestern university. Mean age = 20.2.	Evaluated the role of intrinsic and extrinsic religiousness in modifying family risk on disordered eating among women
Gates et al. (2009) (USA) [36]	Cross-Sectional study	N = 330 undergraduate students at a large state university in the North West. Female (67 %), Male (33 %). Mean age = 21.42. Christian (49 %), Catholic (15 %), Latter Day Saints (12 %), Other religious affiliation not listed (7 %), No religious affiliation (17 %).	Examined the relationship between religious affiliation, religious angst and disordered eating
Gluck et al. (2002) (USA) [39]	Cross-Sectional study	N = 126 females from several different Universities and Colleges in the Northeast. Female undergraduates, less then 27 years old, born in USA, Judaism as religious affiliation, and Caucasian as ethnic identification. Mean age = 20. Orthodox Jews (62 %), Secular Jews (38 %).	Compared body dissatisfaction and disturbed eating behaviours between Orthodox and Secular Jewish women.
Hayman et al. (2007) (USA) [46]	Cross-Sectional study	N = 204 from Freshman academic success course. Women (63 %), Men (37 %). Mean age = 18.17.	Investigated the relationship between spirituality, body image, self-esteem and stress.
Homan et al. (2013) (USA) [45]	Cross-Sectional study	N = 104 female students from a private Christian liberal arts college. Mean age = 20. Extremely religious (4 %), Very religious (62.6 %), Somewhat religious (28.4 %), Not at all religious (5 %).	Tested whether a warm and secure relationship with God was related to positive body image.
Homan et al. (2010) (USA) [28]	Longitudinal study	N = 231 female students from a private Christian liberal arts college. Mean age = 19.2. Protestant (58 %), Other Christian denominations (41 %). Extremely religious (16 %), Very religious (56 %), Somewhat religious (26 %), Not at all religious (2 %).	Explored whether a secure relationship with God would protect young women from pressure to be thin, thin-ideal internalisation, body dissatisfaction and dieting.
Inman et al. (2014) (USA) [26]	Experimental and Cross-Sectional	N = 111 women from a Christian college. Religion was extremely important (83 %).	Experimentally examined whether religious affirming statements buffered against exposure to thing models. Also looked at relationship between religious commitment, general commitment, and body satisfaction.
Inman (2014) (USA) [27]	Experimental and Cross-Sectional	N = 56 men from a Christian college. Religion was extremely important (56 %).	Experimentally examined whether religious affirming statements or strong religious commitment buffered against media threats.
Jacobs-Pilipski et al. (2005) (USA) [33]	Cross-Sectional study	N = 255 female university students. Recruited from 2 public and 2 private west coast universities. Mean age = 20.7. Protestant (29 %), Roman Catholic (24 %), Jewish (11 %), Non-western religions (19 %), Agnostic/Atheist (17 %).	Examined the spiritual and religious (S/R) beliefs and practices of college-age women, and the relationship between body image distress, coping, and S/R.
Kim (2006) (USA) [20]	Cross-Sectional study	N = 546 community sample. Female- 64 %, Male- 33 %. Mean age: Female- 44, Male- 42. Conservative Protestant (28 %), Mainline Protestant (20 %), Catholic (30 %), Other (15 %), Non-religious (6 %).	Examined religion’s relationships with body satisfaction and dieting.
Latzer et al. (2014) (USA) [43]	Cross- Sectional study	N = 102 Modern Orthodox Jewish adolescent females. Ages 17–18.	Explored religious coping and disordered eating pathology amongst Orthodox Jewish adolescent girls.
Latzer et al. (2007) (Israel) [40]	Cross- Sectional study	N = 320 Jewish religious adolescent school girls in the 9^th^-12^th^ grades from a Modern Orthodox national boarding school.	Examined the relation between level of religiosity, grade level, self-esteem, and level of disordered eating-related psychopathology among Modern Orthodox Jewish adolescent girls living in Israel.
Mahoney et al. (2005) (USA) [31]	Cross-Sectional study	N = 289 college students enrolled in a Midwest state university. Female (77.5 %), Male (22.5 %). Mean age = 19.2. Protestant (38 %), Roman Catholic (36 %), Jewish (1 %), Other (11 %), None (14 %).	Examined how the construct of sanctification might be applied to the human body and how such perceptions are tied to lifestyle variables that can compromise or enhance health.
Pinhas et al. (2008) (Canada) [42]	Cross-Sectional study	N = 1130 Females + 1145 Males living in Toronto and attending high school full time. Mean age: Jewish female- 15.8, Non Jewish female- 15.8, Jewish male- 16, Non-Jewish male- 15. Jewish (55 %), Christian (21 %), Eastern (6 %), Muslim (5 %), Mixed (10 %), Other (3 %).	Contrasted the presence and nature of eating attitudes and behaviours in Jewish, compared to non-Jewish adolescents in Toronto. Also looked at the relation between the type of religious observance and disordered eating attitudes and behaviours in Jewish adolescents.
Watkins et al. (2006) (USA) [32]	Cross-Sectional study	N = 809 female college students. 18–20 years (40.5 %), 21–25 years (35.1 %), 26–29 years (9 %), 30 years + (15.5 %).	Measured and examined the relationship between spiritual well-being and binge eating.
Weinberger-Litman et al. (2008) (USA) [44]	Cross-Sectional study	N = 301 Jewish women. Mean age- 19. Orthodox or Modern Orthodox Jewish (76.1 %), Other (23.9 %).	Assessed the influence of religious orientation and spiritual well-being on body dissatisfaction and disordered eating in Jewish women.

**Table 2:** Relevant outcome measures and results of reviewed studies

Author (Year) (Country)	Measures of Disordered Eating and Psychopathology	Measures of Religiosity/Spirituality	QIS* + Grade	Results
**Disordered Eating and Psychopathology**				
Boisvert et al. (2013) (Canada) [38]	EDI- 1 item from each of DT, B, BD	Religiosity- ‘I would describe myself as religious’ (King et al., 2001)	8 IV	**Correlation-** Spirituality and eating disorder symptomatology (r = −.19, *p* < .001).
		Spirituality- SWBS- 2 items from EWB subscale		**ANCOVA-** Spirituality and eating disorder symptomatology (F(1, 497) = 13.74, *p* < .001).
Boisvert et al. (2012) (Canada) [37]	EDI- 1 item from each of DT, B, BD	Religiosity- ‘I would describe myself as religious’. (King et al., 2001)	8 IV	**Correlation-** Eating disorder symptomatology not significantly related to spirituality (r = −.07) or religion (r = .08).
		Spirituality- SWBS- 2 items from EWB subscale		**Path analysis-** 10 % of the variance in EDI was explained by BMI (b = .28, *p* < .001) and body shame (b = .15, *p* < .001). 6 % of the variance in body shame was explained by age (older men had higher EDI) (b = .15, *p* < .001), being Asian (b = .11, *p* < .01) and lower spirituality (b = −.15, *p* < .001). Spirituality had a sig indirect effect on EDI mediated by body shame (b = −.02).
Boyatzis et al. (2006) (USA) [35]	EDI – DT, B	Quest Scale	10 IV	**Correlation-** College freshmen + sophomores- Bulimia: Total Quest (r = .33, *p* < .01), Existential Questioning (r = .29, *p* < .05), Doubting as positive (r = .23, *p* < .10).
				College juniors + seniors- Openness to Change: Bulimia (r = .28, *p* < .10), Drive for Thinness (r = .27, *p* < .10).
Buser (2013) (USA) [47]	BULIT-R	5 RCOPE subscales	13 IV	**Correlation-** Punishing God/Higher power reappraisal andbulimic symptoms (r = .25, *p* < .001). Other subscales not sig.
		Punishing God/Higher power reappraisal, passive religious deferral, active religious surrender, benevolent religious reappraisal/spiritual support and religious focus.		**Hierarchical Regression-** 1st step: BMI + religious affiliation 7.9 % (F(2, 589) = 25.23. *p* < .001), 2^nd^ step: 5 coping scales 5.5 % (F(5, 584) = 7.469, *p* < .001) Cohen’s f^2^ = .06. Punishing God/Higher power reappraisal- (β = .242, *p* < .001).
				**Mediation Analysis-** Punishing God/Higher power reappraisal partially mediated relationship between stress and bulimic symptoms. Sobel Test (z = 2.975, *p* < .01).
Feinson et al. (2012) (Israel) [41]	DEB-SQ	Self-reported religious observance category	9 IV	**ANOVA-** No Sig Differences.
Forthun et al. (2003) (USA) [29]	FAD- GFF subscale	I/E-R	9 IV	**Correlation-** Intrinsic religiousness: Bulimia (r = −.13, *p* < .01), Drive for Thinness (r = −.07, *p* < .05).
	FARS- items selected			**Hierarchical regression + Simple Slope Analyses**
	EDI – DT, B			**Family dysfunction-** B: Family x Intrinsic (B = −0.53, *p* < .01), Family x Extrinsic (B = 0.45, *p* < .10).
				DT: Family x Extrinsic (B = 0.69, *p* < .10).
				Intrinsic buffered association between family and B. Extrinsic made people more vulnerable to B/DT.
				**Parental History-** B: History x Extrinsic (B = 2.29, *p* < .01).
				DT: History x Intrinsic (B = −2.45, *p* < .05), History x Extrinsic (B = 2.98, *p* < .05).
				Intrinsic buffered association between history and DT. Extrinsic made people more vulnerable to B/DT.
Gates et al. (2009) (USA) [36]	EDI- B, DT	MQOS- Religious angst subscale	8 IV	**ANOVA-** Religious Angst: Higher levels of Drive for Thinness (F =1.86, *p* < 0.01), Bulimia (F =1.89, *p* < 0.01).
Gluck et al. (2002) (USA) [39]	EDE-Q	Orthodox Traditions Scale	9 IV	**ANOVA-** secular Jews had higher EDE-Q scores then Orthodox Jews (F[1,125] = 8.5; *P* = .004).
		RIQ		**Chi-square-** Secular women were more likely to use laxatives to control their shape and weight (*χ* ^2^ [1,123] = 5.8, *p* = .02).
				**MLR-** SES + religious grouping: 11 % total EDE-Q (R^2^ = .11, *p* = .001).
Jacobs-Pilipski et al. (2005) (USA) [33]	EDE	13-item self-report measure of spiritual and religious beliefs and practices.	9 IV	**ANOVA-** No significant relationships.
	EDI-2- DT, B			
Latzer et al. (2014) (USA) [43]	EDI	Brief R-COPE	9 IV	**Correlation-** Negative religious coping: total EDI (r = .28, *p* < .01), Drive for Thinness (r = .23 *p* < .05), Ineffectiveness (r = .24, *p* < .05), Maturity (r = .25, *p* < .05), EAT bulimia (r = .22, *p* < .05).
	EAT-26			**Regression-** Negative religious coping (control BMI): total EDI (R^2^ = .19, *p* < .01), Ineffectiveness (R^2^ = .07, *p* < .05), Maturity (R^2^ = .06, *p* < .05), EAT Bulimia (R^2^ = .07, *p* < .05).
Latzer et al. (2007) (Israel) [40]	EDI-2	Level of Religiosity Questionnaire	10 IV	**ANOVA-** Religious observance: (low > Intermediate = high) total EDI (*p* < 0.008), Interpersonal Distrust (*p* < 0.03), Interceptive Awareness (*p* < 0.003), Asceticism (*p* < 0.01), Impulse Regulation (*p* < 0.02) and Social Insecurity (*p* < 0.03)
				**Correlation-** Religiosity Level: EDI-2 total (r = −0.15 *p* < 0.01), Body dissatisfaction (r = −0.14 *p* < 0.01), Ineffectiveness (r = −0.14 *p* < 0.01), Interpersonal distrust (r = −0.11 *p* < 0.01), Impulse regulation (r = −0.17 *p* < 0.01), Social insecurity (r = −0.16 *p* < 0.01)
				**Regression-** Religiosity and total EDI (*p* = .043)
Mahoney et al. (2005) (USA) [31]	Dieting Practices Inventory- Unhealthy Dieting Practices subscale	Manifestation of God in the Body scale.	9 IV	**Partial Correlation** (BMI + gender controlled): Unhealthy dieting practises: Manifestation of God (r = −.12,*p* < .05), Sacred Qualities (r = −.18, *p* < .001) and Global Religiousness (r = −.13, *p* < .05). Binge eating: Sacred Qualities (r = −.14, *p* < .01).
	Binge Eating Scale	Sacred Qualities of the Body scale.		
		Global Religiousness Score		
Pinhas et al. (2008) (Canada) [42]	EAT 26	Self Reported level of observance	9 IV	No sig differences.
Watkins et al. (2006) (USA) [32]	QEWP-R	SWBS- RWB + EWB	11 IV	**ANOVA- SWBS:** None binge- 96.35, Objective binge- 91.78, Binge trait- 86.11 (p ≤ 0.000) (BT < NB, BT < OB) **RWB:** None binge- 46.34, Objective binge- 43.63, Binge trait- 40.93 (p ≤ 0.000) (BT < NB) **EWB**: None binge- 50.09, Objective binge- 48.15, Binge trait- 45.18 (p ≤ 0.000) (BT < NB, BT < OB).
Weinberger-Litman et al. (2008) (USA) [44]	EAT-26	Self-reported level of religious observance	10 IV	**ANOVA- Religious orientation:** EAT total (F = 5.48, *p* = .001), Dieting (F = 3.78, *p* = .01), Bulimia (F = 4.49, *p* = .004), Oral Control (F = 4.45, *p* = .004). Generally lowest symptom severity to highest: Intrinsic, Anti-Religious, Pro-Religious and Extrinsic.
		ROS		**ANCOVA** (control age, BMI, religious observance, anxiety and depression)- all remain sig except oral control.
		SWB		
**Body Image Concerns**				
Boisvert et al. (2013) (Canada) [38]	OBC- 2 items from Body Shame subscale	Religiosity- ‘I would describe myself as religious’ (King et al., 2001)	8 IV	**Correlation-** Spirituality and Body Shame (r = −.21, *p* < .01).
		Spirituality- SWB- 2 items from EWB subscale		**ANCOVA-** Spirituality and Body Shame (F(1, 502) = 21.36, *p* < .001).
Boisvert et al. (2012) (Canada) [37]	OBC- 2 items from Body Shame subscale	Religiosity- ‘I would describe myself as religious’. (King et al., 2001)	8 IV	**Correlation-** Spirituality and Body shame (r = −.16, *p* < .01).
		Spirituality- SWB- 2 items from EWB subscale		**Path analysis-** 10 % of the variance in EDI was explained by BMI (b = .28, *p* < .001) and Body Shame (b = .15, *p* < .001). 6 % of the variance in Body Shame was explained by age (older men had higher EDI) (b = .15, *p* < .001), being Asian (b = .11, *p* < .01) and lower spirituality (b = −.15, *p* < .001). Spirituality had a sig indirect effect on EDI mediated by Body Shame (b = −.02).
Boyatzis et al. (2007) (USA) [25]	BE Scale	Demographic Survey- Self reported importance of religion	10 IIb	**ANOVA-** BE-Appearance: (F(2, 122) = 5.42, *p* = 0.006). Religious group (M = 1.26) increased more than Control group (M = −0.98) (*p* = 0.005).
	BE- Appearance and BE- weight subscales			BE- Weight: no sig differences.
				**ANCOVA-** BMI and pre-test scores as covariates. BE- Appearance: F(2,114) = 4.20, *p* < 0.02 (pre-test scores), F(2, 114) = 5.53, *p* < 0.01 (BMI).
				BE- weight: no sig differences.
Boyatzis et al. (2006) (USA) [35]	EDI – BD	Quest Scale	10 IV	**Correlation-**College freshmen + sophomores- Body Dissatisfaction: Total Quest scores (r = .34, *p* < .01.), Existential Questioning (r = .31, *p* < .05), Doubting as Positive (r = .28, *p* < .05).
	BE scale			College juniors + seniors- Body Esteem and Openness to Change (r = −.31, *p* < .05)
				College graduates- Body dissatisfaction and Existential Questioning (r = −.25, *p* < .10).
Gates et al. (2009) (USA) [36]	EDI- BD	MQOS- Religious angst subscale	8 IV	**ANOVA-** Religious Angst: Higher levels of body dissatisfaction (F (33, 234) = 1.38, *p* = 0.09).
Gluck et al. (2002) (USA) [39]	BSQ	Orthodox Traditions Scale	9 IV	**ANOVA-** Secular women had higher body dissatisfaction then orthodox women (F[1, 125] = 8.0, *p* = .005).
	FRS	RIQ		**Logistic Regression-** Secular students 2x likely to have a fear of becoming fat (odds ratio [Exp (B)] = 2.3, *p* = .05) + 4x likely to be greatly influenced by their shape and weight (odds ratio [Exp (B)] = 3.8, *p* = .001).
				**MLR-** Religious grouping: 6 % total BSQ (R^2^ = .06; *p* < .03).
Hayman et al. (2007) (USA) [46]	OBC	FMS- 20 items	10 IV	**Correlation-** Whole sample**:** No sig. Women: No sig, Men: spirituality and body surveillance (r = −.41, *p* = .001).
	VAS			
	CDRS			
Homan et al. (2013) (USA) [45]	BAOS	AGI	10 IV	**Correlation-** AG- Anxiety: Body Appreciation (r = −.28, *p* < .01), Body Acceptance by others (r = −.22, *p* < .05), Body Surveillance (r = −.33, *p* < .01).
	BAS	1 item ‘How religious are you’		AG- Avoidance: Body Surveillance (r = −.21, *p* < .05).
	OBC- Body Surveillance	1 item frequency of worship attendance		**Regression-** AG- Anxiety: predicted Body Appreciation (B = −.22, *p* = .038) and Body Surveillance (B = −.29, *p* = .008).
				AG-Avoidance: no sig
Homan et al. (2010) (USA) [28]	Perceived Sociocultural Pressure Scale (Stice, n.d.)	AGI	9 IIb	**Correlation-** AGI (Anxiety): Pressure to be thin (r = .22, *p* < .01), Thin-ideal Internalisation (r = .30, *p* < .001), Body Dissatisfaction (r = .25, *p* < .001), Dieting (r = .26, *p* < .001). AGI (Avoidance): Body Dissatisfaction (r = .18, *p* < .001).
	SATAQ- Internalisation subscale			**Regression-** Thin-ideal Internalisation predicted body dissatisfaction in secure and anxious relationship groups (t(222) = 2.31, *p* < .02, R^2^ = .08 vs. R^2^ = .27). Secure relationship buffers this relationship.
	MBSRQ- Body Areas Satisfaction subscale			**Hierarchical regression-** Anxious group- Body Dissatisfaction predicted by Pressure to be Thin (R^2^ = .03, *p* < .05), Thin Ideal Internalisation (R^2^ = .03, *p* < .05). Secure group- not sig.
	DRES			
Inman et al. (2014) (USA) [26]	BE	Demographic Survey- Self reported importance of religion	10 IIb	**ANOVA-** Feelings of love and acceptance between the four statement groups (F(3, 106) = 8.22, *p* < .001). Religious (Mnonbody = 7.59, Mbody = 7.41) greater then Non-Religious (Mcontrol = 5.22, Mpositive = 6.73) (t(106) = 4.08, *p* < .001).
	EDI- DT, BD	GCS		**2 way ANCOVA-** Appearance Esteem between the different statement groups not sig (F(3, 101) = .29, *p* = .83). Affirmation group x religious commitment interaction (F(3, 101) = 2.99, *p* < .04, η2 = .08).
	RDS	RCS		Weight esteem between the different statement groups not sig (F(3, 101) = .52, *p* = .60), Affirmation group x religious commitment interaction (F(3, 101) = 3.42, *p* < .03, η2 = .09).
		Emotions		**Correlation-** Religious Commitment: Appearance Esteem (r = .20, *p* < .05), Weight Esteem (r = .19, *p* < .05), Body Dissatisfaction (r = −.21, *p* < .05).
				**Partial Correlation** (control for general commitment)- Religious commitment: Body Dissatisfaction (r = −.20, *p* < .05).
Inman (2014) (USA) [27]	BE	RCS	10 IIb	**ANOVA-** no sig differences between groups.
		Emotions		**2 way ANOVA-** religiously committed men had higher Appearance Esteem at time 1 then less religious men (F(1, 48) = 17.76, *p* = .001, η2 = .27.). No sig interactions.
				**Correlation-** Religious commitment: higher Appearance (r = .34, *p* < .05) and Weight Esteem (r = .35, *p* < .01).
				**Hierarchical regression and simple slope analysis:** Religious commitment moderated the effect of harmful media on Weight Esteem in heavy men.
Jacobs-Pilipski et al. (2005) (USA) [33]	EDI-2- BD	Brief COPE	9 IV	**ANOVA-** No significant relationships between S/R beliefs/practices and Body Dissatisfaction.
		13-item self-report measure of spiritual and religious beliefs and practices.		**T- Test-** Women with strong S/R beliefs/practices more likely to read religious works (T = 7.3, *p* = .000), pray (T = 9.3, *p* = .000) and meditate (T = 3.8 *p* = .007) than women without strong S/R beliefs/practices.
				Women with strong S/R beliefs/practices less likely to use distraction to cope with body image distress (T = 2.7, *p* = .007) and reported prayer to be an effective strategy for dealing with dissatisfaction with weight and shape (T = 3.0, *p* = .004).
Kim (2006) (USA) [20]	EDI- BD	Compilation of measures- religious practice, application, commitment, identity, coping, social support	11 IV	**Regression (**controlling BMI + demographics).
				**Men-** Body satisfaction: positive spiritual coping (beta = 0.18, *p* < 0.05), negative spiritual coping (beta = −0.58, *p* < 0.01). Closer relationship with God (beta = 0.14, *p* < 0.05). All religious variables (R^2^ = 0.174, *p* < 0.05).
				**Women-** Body satisfaction: positive spiritual coping (beta = 0.19, *p* < 0.01), negative spiritual coping (beta = −0.47, *p* < 0.01), spending more hours on religious and spiritual activities (beta = 0.01, *p* < 0.01), prayer (beta = 0.23, *p* < 0.05). All religious variables (R^2^ = 0.106, *p* < 0.01).
Mahoney et al. (2005) (USA) [31]	MBSRQ- Body Areas Satisfaction and Appearance Orientation subscales	Manifestation of God in the Body scale.	9 IV	**Partial Correlation** (BMI + gender controlled)- Body area satisfaction: Manifestation of God (r = .13,*p* < .05), Sacred Qualities (r = .25, *p* < .001) and Global Religiousness (r = .13, *p* < .05).
		Sacred Qualities of the Body scale.		**Hierarchical regression-** Body area satisfaction- Step 1: Race, gender, global religiousness (R^2^ Change = .05, *p* < .01), Step 2: Manifestation of God, Sacred Qualities (R^2^ Change = .04, *p* < .01).
		Global Religiousness Score		
Weinberger-Litman et al. (2008) (USA) [44]	BSQ	Self-reported level of religious observance	10 IV	**ANOVA- Religious Orientation:** Body Satisfaction (F = 6.15, *p* < .001). Intrinsic > extrinsic (*p* = .01), pro-religious (*p* < .001). **ANCOVA-** (control age, BMI, religious observance, anxiety and depression)- all remain sig
		ROS		**ANOVA- Spiritual Well-Being:** High > Moderate (F = 4.57, *p* = .03, d = .27) Body Satisfaction. **Existential Well-Being:** High > Moderate (F = 18.23, *p* < .001, d = .51) Body Satisfaction. **ANCOVA-** (control age, BMI, religious observance)- all remain sig, (control anxiety and depression)- loose sig.
		SWB		

*QIS- Quality Index Score

Abbreviations

AGI- Attachment to God Inventory

BAOS- Body Acceptance by Others Scale

BAS- Body Appreciation Scale

BE- Body Esteem Scale

Binge Eating Scale

Brief COPE

BSQ- Body Shape Questionnaire

BUILT-R- Bulimia Test Revised

CDRS- Contour Drawing Rating Scale

DEB-SQ- Disordered Eating Behaviors—Screening Questionnaire

Dieting Practices Inventory

DRES- Dutch Restrained Eating Scale

EAT-26- Eating Attitude Test

EDE- Eating Disorder Examination

EDE-Q- Eating Disorders Examination- Questionnaire Version

EDI- Eating Disorder Inventory, DT- Drive for Thinness, B- Bulimia, BD- Body Dissatisfaction

EDI-2- Eating Disorder Inventory-2

FAD- McMaster Family Assessment Device, GFF- General Family Functioning

FARS- Family Addiction and Recovery Scale

FMS- Faith Maturity Scale

FRS- Figure Rating Scale

GCS- General Commitment Scale

Global Religiousness Score

I/E-R- Intrinsic/Extrinsic-Revised scale

Level of Religiosity Questionnaire

Manifestation of God in the Body scale and Sacred Qualities of the Body scale

MBSRQ- Multidimensional Body-Self Relations Questionnaire

MQOS- Multidimensional Quest Orientation Scale

OBC- Objectified Body Consciousness scale

PACS- Physical Appearance Comparison Scale

QEWP-R- The Questionnaire on Eating and Weight Patterns-Reviseds

Quest Scale

RCOPE- Religious Coping Questionnaire

RCS- Religious Commitment Scale

RDS- Restrictive dieting scale

RIQ- Religious Identification Questionnaire

ROS- Religious Orientation Scale

SATAQ- Sociocultural Attitudes Towards Appearance Questionnaire

SCSRFQ- Santa Clara Strength of Religious Faith Questionnaire

SOQ- Self-Objectification Questionnaires

SWBS- Spiritual Well-Being scale. EWB- Existential Wellbeing Subscale, RWB- Religious Well-Being

VAS- Weight and Appearance Visual Analogue Scales

## Results

### Description of studies

Twenty-two studies met our inclusion criteria. There were four articles published from 2001–2005, 10 from 2006–2010, and eight from 2011–2014. Fifteen included only female participants, two only male participants and five both genders. With regard to the sample of participants, 13 recruited participants from American universities or colleges, six recruited from Jewish communities, and three recruited from other sources within the community. The majority of studies were based in the USA, with others coming from Israel and Canada.

### Quality assessment

With a possible total score of 14, studies reported a range from 8–12, with a mean of 9.55. The reader is directed to Appendix 6 for the quality assessment of the included studies.

**Table 3:** Quality index of included studies (Modified from Ferro & Speechley, 2009)

Author (year)	Hypothesis Clearly Described	Main outcomes clearly described	Characteristics of participants described	Main findings clearly described	Estimates of Random Variability	Actual Probability Values Used	Response Rate Clearly Described	Participants Representative of Population	Final Sample Representative of Population	Statistical Tests Appropriate	DE, P, BIC measures valid/reliable	S/R measures valid/reliable	Adjustment for Confounding	SampleSize or Power Calculation	Total Index Score
Boisvert et al. (2013) [38]	1	1	1	1	1	0	0	1	0	1	0	0	1	0	8
Boisvert et al. (2012) [37]	1	1	1	1	1	0	0	1	0	1	0	0	1	0	8
Boyatzis et al. (2007) [25]	1	1	1	1	1	1	0	0	0	1	1	1	1	0	10
Boyatzis et al. (2006) [35]	1	1	1	1	1	0	1	1	0	1	1	1	0	0	10
Buser et al. (2013) [47]	1	1	1	1	1	1	1	1	0	1	1	1	1	1	13
Feinson et al. (2012) [41]	1	1	1	1	1	0	0	1	0	1	1	0	1	0	9
Forthun et al. (2003) [29]	1	1	1	1	1	0	0	0	0	1	1	1	1	0	9
Gates et al. (2009) [36]	1	1	1	1	1	0	0	0	0	1	1	1	0	0	8
Gluck et al. (2002) [39]	1	1	1	1	1	1	0	0	0	1	1	0	1	0	9
Hayman et al. (2007)[46]	1	1	1	1	1	1	1	0	0	1	1	1	0	0	10
Homan et al. (2013) [45]	1	1	1	1	1	1	0	0	0	1	1	1	1	0	10
Homan et al. (2010) [28]	1	1	1	1	1	0	0	0	0	1	1	1	1	0	9
Inman et al. (2014) [26]	1	1	1	1	1	1	0	0	0	1	1	1	1	0	10
Inman (2014) [27]	1	1	1	1	1	1	0	0	0	1	1	1	1	0	10
Jacobs-Pilipski et al. (2005) [33]	1	1	1	1	1	1	0	0	0	1	1	1	0	0	9
Kim (2006) [20]	1	1	1	1	1	0	1	1	0	1	1	1	1	0	11
Latzer et al. (2014) [43]	1	1	1	1	1	0	0	0	0	1	1	1	1	0	9
Latzer et al. (2007) [40]	1	1	1	1	1	0	0	1	1	1	1	0	1	0	10
Mahoney et al. (2005) [31]	1	1	1	1	1	0	0	0	0	1	1	1	1	0	9
Pinhas et al. (2008) [42]	1	1	1	1	1	1	0	0	0	1	1	0	1	0	9
Watkins et al. (2006) [32]	1	1	1	1	1	1	1	1	0	1	1	1	0	0	11
Weinberger-Litman et al. (2008) [44]	1	1	1	1	1	1	0	0	0	1	1	1	1	0	10

The mean subscale score was 5.73/7.0 (range 5–7) for reporting, with the majority of studies having well described hypotheses, participant characteristics, outcomes and results. However less than half of the papers reported actual probability values, while the majority gave no indication of response rates. The mean subscale score was 0.41/2.0 for external validity (range 0–2), with the majority of authors making use of convenience sampling methods that limited representation as opposed to randomised recruitment strategies used by a minority of studies. Furthermore, there were no comparisons made between responders and non-responders.

The mean subscale score was 3.41/4.0 for internal validity (range 2–4). While many papers made use of valid and reliable measures for disordered eating, psychopathology and body image concerns, the reliability and validity of religious and spiritual measures were of concern. However, the majority of papers adequately adjusted for confounding in their analysis. Power calculations were rarely included in the vast majority of studies.

According to the hierarchy of evidence, only four studies were graded as providing fairly strong evidence. These were three experimentally-designed studies [25–27] and one longitudinal study [28], all exploring body image concerns. In contrast, the other 18 studies were classified as providing weak evidence due to the cross-sectional design of the studies.

### The relationship between aspects of religiosity, spirituality and disordered eating, psychopathology and body image concerns

Of the 22 studies that were reviewed, seven assessed disordered eating and psychopathology, seven assessed body image concerns and eight assessed both concepts.

### Disordered eating and psychopathology

A total of 15 articles explored the relationships between aspects of religiosity, spirituality and disordered eating and psychopathology in non-clinical samples.

### American University/College samples

Researchers have explored concepts which pertain to religious and spiritual beliefs along with religious anxiety and doubt in student populations. The results reflect a mixture of positive and negative associations with disordered eating and psychopathology.

Providing insight about the depth of religious belief, whether intrinsic or extrinsic, Forthun and colleagues (2003) [29] examined the moderating effects of religious orientation on the relationship between family risk and disordered eating in women. An intrinsic believer is someone who is devout and motivated to live his or her religion due to internalised personal beliefs [30]. On the other hand, an extrinsic believer pursues religion for social reasons, and views it as a way of achieving status, acceptance and security [30]. Through a hierarchical regression model and simple slope analysis, intrinsic religiousness was shown to buffer the association between family dysfunction and bulimic symptomatology. Similarly, In relation to a parental history of disordered eating, intrinsic religiousness reduced the association between parental history and drive for thinness.

Specific religious beliefs related to the body were explored by Mahoney and colleagues (2005) [31] in a sample of women and men. These beliefs included the perception of the body being a manifestation of God, and having divine and sacred qualities. Partial correlations, while controlling for gender and race resulted in unhealthy dieting practices being weakly correlated with lower levels of manifestation of God (r = −.12,*p* < .05) and sacred qualities (r = −.18, *p* < .001). Higher levels of binge eating were also related to lower levels of sacred qualities (r = −.14, *p* < .01). Similarly, Watkins and colleagues (2006) [32] discovered that females with greater binge-eating severity had significantly lower spiritual and existential well-being scores (*p* < 0.000) compared to those that displayed lower levels of binge eating.

In contrast to the studies above, Jacobs-Pilipski and colleagues (2005) [33] found no differences relating to disordered eating or psychopathology between two groups of women who were divided based on strength of religious beliefs and practices. The lack of significance may have been due to the exclusive use of women with elevated levels of weight and shape concern, along with alpha having been set to .01. Negative associations were also found between certain religious beliefs and disordered eating and psychopathology. Extrinsic religiosity was shown to make students with greater family dysfunction or a parental history of disordered eating more vulnerable to bulimic symptomatology and drive for thinness [29]. Additionally, the belief in a punishing God/higher power used to cope in times of difficulty was associated with increased bulimic behaviour (r = .25, *p* < .001) [34].

Religious anxiety and doubt have also been linked with higher levels of disordered eating and psychopathology. Boyatzis and McConnell (2006) [35] discovered that greater religious uncertainty was moderately correlated with bulimic symptomatology in female college freshmen and sophomore students (r = .33, *p* < .01). Existential questioning (r = .29, *p* < .05) and doubting being seen as positive (r = .23, *p* < .10) were also related to bulimic symptomatology, with smaller effect sizes. Furthermore, Gates and Pritchard (2009) [36] revealed that religious angst as a result of doubt and anxiety with one’s experience of religion was significantly associated with bulimic symptomatology (*p* < 0.01) and drive for thinness (*p* < 0.01), however there were no measures of effect size given.

### Western Community samples

Studies examining community samples have explored self-rated levels of religiosity and spirituality, with spirituality being linked to lower levels of disordered eating.

Unlike the previous research presented until now, Boisvert and Harrell (2012) [37] set out to test a well researched path model that included ethnicity, spirituality, religiosity, body shame, BMI and age as risk factors for disordered eating in a sample of men. Disordered eating was not significantly related to spirituality (r = −.07) or religion (r = .08). However, regression analyses were used to test the predicted path model. Ten percent of the variance in disordered eating was explained by BMI (*p* < .001) and body shame (*p* < .001) and 6 % of the variance in body shame was explained by age, Asian ethnicity and lower spirituality (*p* < .001). The authors argue that spirituality had an indirect effect on disordered eating mediated by body shame in their male sample. Additionally, Boisvert and Harrell (2013) [38] discovered a small relationship between spirituality and lower levels of disordered eating (r = −.19, *p* < .001) in a sample of women, with the result maintaining significance while controlling for body shame, BMI and age (*p* < .001).

### Jewish samples

The Jewish population is a useful sample to research due to its distinct religious groups with varying degrees of religious observance. Thus researchers have compared disordered eating and psychopathology between observance groups, with positive or non-significant results. Religious coping and orientation have also been explored in Jewish populations.

In a sample of Jewish women living in America, Gluck and Geliebter (2002) [39] reported that secular Jewish women displayed greater disordered eating psychopathology then their orthodox counterparts (*p* = .004). However the mean scores for each group and effect sizes were not provided. Latzer and colleagues (2007) [40] found similar results while investigating a sample of modern orthodox Jewish adolescent girls in an Israeli boarding school. Total Eating Disorder Inventory-2 (EDI-2) scores were significantly higher (*p* < 0.008) in the low religious observance group (63.7) compared to the intermediate (47.5) and high observance groups (43.6). Comparable results were obtained for five subscales of the EDI-2. There was also a small correlation between higher levels of religiosity and lower scores on the EDI-2 (r = −0.15, *p* < 0.01) and five of its subscales.

While these two studies have found positive associations between greater religious observance and lower levels of disordered eating and psychopathology, the following two studies failed to do so. Feinson and Meir (2012) [41] made use of random recruitment of women in Israel for a more representative sample. However, there were no significant differences between ultra-orthodox, orthodox, traditional and secular Jews with regards to disordered eating. Furthermore, Pinhas and colleagues (2008) [42] observed the same effect surveying female Jewish high school students in Canada.

While religious observance seemed to be the mainstay for research relating to Jewish samples, two studies explored religious coping and orientation. Examining modern orthodox Jewish adolescent females living in New York, Latzer and colleagues (2014) [43] discovered that negative religious coping was weakly associated with higher total EDI scores (r = .28, *p* < .01) and increased bulimic symptomatology (r = .22, *p* < .05). With a similar sample, Weinberger-Litman and colleagues (2008) [44] established that Jewish women with an intrinsic religious orientation had less disordered eating then those with an extrinsic religious orientation and that an extrinsically orientated person had more disordered eating than someone who was non-religious, or showed no orientation bias. Thus both studies highlight the multifaceted nature of religiosity and potential adverse associations with disordered eating.

### Body image concerns

A total of fifteen articles explored the relationships between aspects of religiosity, spirituality and body image concerns in non-clinical samples.

### American University/College samples

Research into religiosity, spirituality and body image concerns has centred around the stability and security of a relationship with God along with religious strategies and beliefs that may help people cope with body dissatisfaction,

With religious questioning (r = .31, *p* < .05) and doubt (r = .28, *p* < .05) being moderately linked with greater body dissatisfaction [35], the concept of a warm and secure relationship with God was explored in regards to body image. Homan and Cavanaugh (2013) [45] revealed that a less anxious relationship with God was weakly correlated with higher levels of body appreciation (r = −.28, *p* < .01) and moderately correlated with lower levels of body surveillance (r = −.33, *p* < .01). Regression analyses, while controlling for parental attachments, confirmed these predictions.

Homan and Boyatzis (2010) [28] expanded on this concept through use of a longitudinal study design. Results revealed that an anxious relationship with God produced small correlations with greater body dissatisfaction (r = .25, *p* < .001) and thin-ideal internalisation (r = .30, *p* < .001). It was also discovered that a secure relationship with God buffers the effects of thin-ideal internalisation on body dissatisfaction (*p* < .02, R^2^ = .08 vs. R^2^ = .27). In addition, hierarchical regression analysis, while controlling for baseline scores, revealed that women who had a warm and secure relationship with God were protected over time from the effects of social and cultural pressures on body image. Thus the results provide evidence of directionality and protection against body image concerns.

Further studies explored the effects of religious strategies used to cope with body dissatisfaction. Jacobs-Pilipski and colleagues (2005) [33], using a sample of women with elevated weight and shape concerns, discovered that women with strong beliefs and practices were more likely to read religious works (*p* = .000), pray (*p* = .000) and meditate (*p* = .007) than participants without strong beliefs and practices. Prayer was the only reported strategy to be significantly effective for those with strong beliefs, compared to those without (*p* = .004).

Adding considerable strength to the evidence were two similar studies which adopted an elegant experimental design in assessing the use of religious material. Boyatzis and colleagues (2007) [25] conducted a study using a Random Assignment, pretest-posttest design to prove that reading religious messages about one’s body improves women’s body image. A sample of 125 women completed a demographic survey and a pretest body esteem scale with weight and appearance subscales. The participants were assigned via matched random assignment to one of three body affirmation condition groups. The strength of this study emanates from the matching process, with each group having similar body esteem, BMI and religiosity scores.

 One week later, the control group read through 15 statements about university events, with no religious or spiritual aspects. The religious group read Christian-based body affirmation statements which were explicitly theistic, while the spiritual group read similar body affirmations, but with no reference to God. Distractor tasks were then used, and participants spent time looking at photographs of fashion models epitomising the ‘thin ideal’ before completing the post test body esteem scale. A one-way ANOVA comparing the change scores revealed a significant difference between group means (*p* = 0.006). Women who read religious body affirmations (M = 1.26) felt better about their appearance compared to the control group (M = −0.98), who felt worse (*p* = 0.005). These results were confirmed while controlling for pre-test scores (*p* < 0.02) and BMI (*p* < 0.01).

 This article, to our knowledge, was the first to provide experimental evidence that reading religious messages offers some protection against body image concerns. The messages promoted divine acceptance and a balanced perspective and seemed to offer protection against the allure images that promoted a thin figure. The results reflect the work of Mahoney et al. (2005), in which viewing one’s body as having sacred qualities (r = .25, *p* < .001) and being a manifestation of God (r = .13, *p* < .05) were linked with higher levels of body satisfaction [31]. The causal link that this article provides is an improvement on the limitations on the numerous cross-sectional studies available in this field. However, the article could be improved with reporting of Eta squared or cohen’s d values for a better judgment of effect size. The study also needs to be replicated with a broader diversity of participants with regards to religiosity and ethnicity.

Inman and colleagues (2014) [26] expanded on the experimental work of Boyatzis et al. (2007) [25] by looking at religious body affirming statements, body image concerns and religious commitment in 111 women. The measures and method of the experimental component of the study were similar to those of Boyatzis et al. (2007) [25]. In contrast, changes in appearance esteem were not significant between the different groups (*p* = .83). However religiously-committed women generally showed a greater increase in appearance and weight esteem compared to women with low religious commitment in most groups. This could possibly be attributed to their already internalised beliefs. Religious commitment was weakly related to appearance esteem (r = .20, *p* < .05), weight esteem (r = .19, *p* < .05), and reduced body dissatisfaction (r = −.21, *p* < .05), but only body dissatisfaction (r = −.20, *p* < .05) remained significant when removing the effect of general commitment through partial correlation.

These two studies provide stronger evidence for improving body image concerns through reading religious material and greater religious commitment. The different results obtained in this study when compared to Boyatzis et al. (2007) [25] may be attributable to the characteristics of the participants, with the self-rated importance of religion considerably higher in the Inman et al. (2014) [26] sample (83 %) compared to the Boyatzis et al. (2007) [25] study (39 %).

While most researchers have exclusively sampled women, two studies have explored body image concerns in men. In a separate paper, Inman (2014) [27] found no significant differences in weight or appearance esteem between the differing body affirmation groups (*p* = .50). However, religious commitment was moderately related to higher appearance (r = .34, *p* < .05) and weight esteem (r = .35, *p* < .01) and also moderated the effects of harmful media on weight esteem in heavy men. The link was mediated by transcendent emotions such as peace and assurance. Furthermore, Hayman and colleagues (2007) [46] revealed that men who embraced personal faith in a life-altering manner to a greater degree were less concerned about constant monitoring of their bodies (r = −.41, *p* = .001).

### Western Community samples

Three studies surveyed community populations, examining spirituality and various aspects of religiosity. Boisvert and Harrell (2013) [38] revealed a small relationship between spirituality and lower levels of body shame (r = −.21, *p* < .01), which was confirmed (*p* < .001) while controlling for ethnicity and BMI. In a separate study on men, Boisvert and Harrell (2012) [37] also discovered a small relationship between spirituality and lower levels of body shame (r = −.16, *p* < .01).

Kim (2006) [20] examined the relationship between religion and body image in a community sample recruited from various religious groups and community settings in New York. Regression analyses were run, with body satisfaction being regressed against particular religious variables while controlling for BMI, age, race and education. For men, positive spiritual coping mechanisms used in difficult times, as well as a closer relationship with God predicted greater body satisfaction (*p* < 0.05), while negative spiritual coping predicted lower body satisfaction (*p* < 0.01). It was found that self-esteem played a mediating role between religiosity and body satisfaction. For women, positive spiritual coping (*p* < 0.01), spending more hours on religious and spiritual activities (*p* < 0.01) and prayer (*p* < 0.05) all predicted greater body satisfaction (*p* < 0.01), while negative spiritual coping predicted lower body satisfaction (*p* < 0.01). Self-esteem was also seen to mediate the relationship between these religious aspects and body satisfaction.

### Jewish samples

While there is a growing amount of literature on disordered eating and psychopathology in Jewish populations, only two papers were identified which looked at body image concerns. Gluck and Geliebter (2002) [39] demonstrated that secular Jewish women had greater body dissatisfaction then orthodox women (*p* = .005), were twice as likely to have a fear of becoming fat (*p* = .05), and four times more likely to be greatly influenced by their shape and weight (*p* = .001). These results persisted whilst controlling for SES and media exposure.

Taking a different approach, Weinberger-Litman and colleagues (2008) [44] suggested that Jewish women with an intrinsic orientation displayed greater body satisfaction compared to women with an extrinsic orientation (*p* < .01). These results remained significant after controlling for age, BMI, religious observance, anxiety and depression. Exploring spirituality, participants with high spiritual well-being had greater body satisfaction then those with moderate levels of spiritual well-being (d = .27, *p* = .033). According to cohen’s d, this was a small effect size. Similarly, participants with a high existential well-being had greater body satisfaction than those with moderate levels of existential well-being (d = .51, *p* < .001), with a moderate effect size. These results remained significant when controlling for demographic variables.

## Discussion

The aim of this systematic review was to critically examine the existing literature that has reported on the relationship between aspects of religiosity, spirituality and disordered eating, psychopathology, and body image concerns. Of the 15 articles which investigated religiosity, spirituality, disordered eating and psychopathology, six displayed positive relationships, four demonstrated negative relationships, two revealed both positive and negative relationships, while three revealed no links at all. Thus measurable aspects of religiosity and spirituality had either a positive or negative relationship with disordered eating and psychopathology. This was not the case with body image, however, where 80 % of the articles reviewed reported positive associations with aspects of religiosity and spirituality.

Overall, the main findings to emerge were that strong and internalised religious beliefs coupled with having a secure and satisfying relationship with God were associated with lower levels of disordered eating, psychopathology and body image concern. The religious beliefs were characterised as being intrinsic and devout in nature, with deeply internalised beliefs manifested through strong religious observance and commitment [30]. Specific beliefs consisted of the body having sacred qualities and being a manifestation of God. Furthermore, there was strong evidence to suggest the efficacy of religious beliefs as coping strategies by means of prayer and reading body-affirming religious material. The quality of one’s relationship with God was also found to be equally important. A positive relationship was portrayed as being close, warm and secure, and one which exhibited low levels of anxiety and angst.

Conversely, a superficial faith coupled with a doubtful and anxious relationship with God were associated with greater levels of disordered eating, psychopathology and body image concern. A superficial faith was characterised by an extrinsic religious orientation, with religion being pursued for social reasons, and seen as a way of achieving status, acceptance and security. Beliefs were loosely held to serve other interests and lacked internalisation [30]. Doubting and questioning of religious beliefs reflected greater religious uncertainty, while religious worry, angst and negative coping strategies, such as the belief in a punishing God, contributed to an anxious relationship with God.

 The studies reviewed had a number of evident limitations in design and methodology. Firstly, the vast majority of studies were correlational in design and had small to moderate effect sizes. Thus conclusions about causation and directionality could not be fully established. Only four studies of note were graded as providing fairly strong evidence including Boyatzis et al. [25], Inman et al. [26] and Inman [27] who all made use of a similar experimental design to look at body image concerns. Additionally, the work of Homan and Boyatzis [28] made use of a longitudinal design which confirmed the directionality of the effect.

 The methods used to recruit participants in the majority of the studies could have lead to sampling bias. Many researchers made use of convenience sampling, having sent e-mails and advertising for participation in educational settings. This could have resulted in more religious and spiritual participants taking part out of interest, leading to a less representative sample. The charecteristics of those who did not choose to participate were not identified in most articles, and without the process of randomisation, possible confounding factors could have affected the results. Sample size also ranged in number, with a few studies making use of a small number of participants. Moreover, the limited generalisability of the samples was also a major limitation, due to samples primarily consisting of ethnically white and religiously Christian or Jewish students. Further work needs to be done on samples with a wider range of ages, ethnicities and religious or spiritual identities.

 Although the majority of studies made use of existing valid and reliable measures, some authors either constructed their own surveys or made use of certain questions from known measures in isolation from the rest of the scale. Thus the validity and reliability of these measures are questionable. Most measures also contained a heavy Judeo-Christian bias, which limits their use to participants of those faiths. Additionally, the multifaceted nature of religiosity and spirituality naturally lead to a wide variety of measures used to assess these concepts. While it is important to explore the effects of these facets, it precludes the opportunity for inter-study comparison of results. This review also echoes the sentiments of Boyatzis and Quinlan [22] regarding the limitation of self-report measures, and the need for multiple data sources and qualitative data.

This systematic review aimed to update and expand upon the work of Boyatzis and Quinlan [22]. A recent surge in published literature has lead to new insights on the topic, including a greater utilisation of Jewish samples, allowing for between-group analyses. While many of the conclusions remain the same, this review discovered increased evidence for the potential negative effects of religiosity and spirituality. By also including samples of men in this review, the above results can be translated with greater heuristic value into the wider community.

### Limitations of current study

Although this review had several strengths including the adoption of the PRISMA methodology, there were a number of limitations. While the search strategy made use of an extensive list of search terms, relevant articles may have been omitted due to the multidimensional nature of the topic. In fact two additional papers were identified through peer-review [47, 48]. We therefore recommend future reviews to expand on the search terms used. Another potential limitation of this systematic review was the omission of a meta-analysis. There are often difficulties in conducting a meta-analysis of different outcomes, however it is possible to pool data using different instruments measuring similar religious and spiritual constructs [49, 50]. We therefore recommend that any future papers in this area undertake a meta-analysis. Furthermore, although the review only included articles which made use of non-clinical samples, it is entirely possible that some patients with a clinical presentation may have been inadvertently included in the papers reviewed.

### Conclusion and Future directions

Since there were methodological limitations in the studies reviewed, it is imperative that future research adopt more rigorous research designs. Further use of experimental and longitudinal designs would allow for the exploration of causality and directionality. There also needs to be a greater focus on the mechanisms by which religiosity and spirituality influence disordered eating, psychopathology and body image concerns through mediation and moderation analyses. Valuable insight into the protective role of religiosity and spirituality provides stronger evidence for use in community settings.

Additionally, the use of randomised sampling methods is needed to improve representativeness, which in turn will help to reduce confounding. Greater focus is needed on recruiting ethnically and religiously diverse samples and possible collaboration with universities in Asia, Africa and the Middle East in future research is recommended. In saying that, standardised measures of religiosity and spirituality need to be developed which can be administered across ethnic and religious lines. This would allow for comparison and meta-analysis which would strengthen the evidence in this field.

Finally, diseases such as anorexia nervosa remain difficult to treat, especially in adults and new paradigms need to be explored. Such paradigms would apply to primary and secondary prevention as well as contribute to effective therapeutic regimens. There is evidence to suggest that religious and spiritual interventions may be effective in the treatment of eating disorders [51]. There already appears to be some evidence that enhancing self-esteem and developing a more positive relationship with ones body may offer some protection against developing an eating disorder [52]. If further studies can demonstrate the protective benefits of religiosity and spirituality in the development of a clinical eating disorder, then a stronger evidence base can be utilised to develop community awareness and programs that have the potential to reduce the risk of developing a full-blown eating disorder. It is hoped that this review of non-clinical, community samples will form the basis for such further studies.

## Flow diagram of the article exclusion process

ᅟ

## Appendix 2

### Modified quality index used to assess included studies (Modified from Ferro & Speechly, 2009)

**Reporting**

1. Is the hypothesis/objective of the study clearly described?
2. Are the main outcomes to be measured clearly described in the Introduction or Methods section?
3. Are the characteristics of the patients included in the study clearly described?
4. Are the main findings of the study clearly described?
5. Does the study provide estimates of the random variability in the data for the main outcome?
6. Have actual probability values been reported for the main outcomes except where the probability value is less than 0.001?
7. Is the response rate clearly described?

**External validity**

8. Were the patients asked to participate in the study representative of the entire population from which they were recruited?
9. Were patients who were prepared to participate representative of the entire population from which they were recruited?

**Internal validity (bias and confounding)**

10. Were the statistical tests used to assess the main outcomes appropriate?
11. Were disordered eating, psychopathology or body image concern measures used valid and reliable?
12. Were religiosity or spirituality measures used valid and reliable?
13. Was there adequate adjustment for confounding in the analyses from which the main results were drawn?

**Power**

14. Did the study provide a sample size or power calculation to detect important effects where the probability value for a difference being due to chance is less than 0.05?

## Appendix 3

### Grading criteria (Becker et al., 2007 [24], Modified from Clinical Guidance Outcomes Group.)

**Grade I (strong evidence)**

RCTs or review of RCTs

- **○ IA** Calculation of sample size and accurate and standard definition of appropriate outcome variables
- **○ IB** Accurate and standard definition of appropriate outcome variables
- **○ IC** Neither of the above

**Grade II (fairly strong evidence)**

Prospective study with a comparison group (non-randomised controlled trial, good observational study or retrospective study that controls effectively for confounding variables)

- **○ IIA** Calculation of sample size and accurate, standard definition of appropriate outcome variables and adjustment for the effects of important confounding variables
- **○ IIB** One or more of the above

**Grade III (weaker evidence)**

Retrospective or observational studies

- **○ IIIA** Comparison group, calculation and sample size, accurate and standard definition of appropriate outcome variables
- **○ IIIB** Two or more of the above
- **○ IIIC** One or none of these*

**Grade IV (weak evidence)**

Cross sectional study, Delphi exercise, consensus of experts

## Summary of reviewed studies

ᅟ

## Relevant outcome measures and results of reviewed studies

ᅟ

## Quality index of included studies (Modified from Ferro & Speechley, 2009)

ᅟ

## Abbreviation

EDI: Eating Disorder Inventory
